# Radiologist-Validated Automatic Lumbar T1-Weighted Spinal MRI Segmentation Tool via an Attention U-Net Algorithm

**DOI:** 10.3390/diagnostics15233046

**Published:** 2025-11-28

**Authors:** Aryan Kalluvila, Ethan Wang, Michael C Hurley, Colbey Freeman, Jason M. Johnson

**Affiliations:** 1 Weinberg College of Arts and Sciences, Northwestern University, Chicago, IL 60201, USA; 2 The University of Texas Southwestern Medical School, Dallas, TX 75390, USA; ethan.wang@utsouthwestern.edu; 3 Department of Radiology, University of Chicago, Chicago, IL 60637, USA; mhurley@bsd.uchicago.edu; 4 Department of Radiology, University of Pennsylvania Health System, Philadelphia, PA 19104, USA; 5 Section of Neuroradiology, Yale New Haven Hospital, New Haven, CT 06510, USA; jason.johnson@yale.edu

**Keywords:** artificial intelligence (AI), T1-weighted MRI (T1-w), machine learning (ML), structural similarity index (SSIM)

## Abstract

**Background/Objectives:** Spinal MRI segmentation has become increasingly important with the prevalence of disc herniation and vertebral injuries. Artificial intelligence can help orthopedic surgeons and radiologists automate the process of segmentation. Currently, there are few tools for T1-weighted spinal MRI segmentation, with most focusing on T2-weighted imaging. This paper focuses on creating an automatic lumbar spinal MRI segmentation tool for T1-weighted images using deep learning. **Methods:** An Attention U-Net was employed as the main algorithm because the architecture has shown success in other segmentation applications. Segmentation loss functions were compared, focusing on the difference between BCE and MSE loss. Two board-certified radiologists scored the output of the Attention U-Net versus four other algorithms to assess clinical relevance and segmentation accuracy. **Results:** The Attention U-Net achieved superior results, with SSIM and DICE coefficients of 0.998 and 0.93, outperforming other architectures. Both radiologists agreed that the Attention U-Net segmented lumbar spinal images with the highest accuracy on the Likert Scale (3.7 ± 0.82). Cohen’s Kappa coefficient was measured at 0.31, indicating a fair level of agreement. MSE loss outperformed BCE with respect to both SSIM and DICE, serving as the loss function of choice. **Conclusions:** Qualitative observations showed that the Attention U-Net and U-Net++ were the top performing networks. However, the Attention U-Net minimized external noise and focused on internal spinal preservation, demonstrating strong segmentation performance for T1-weighted lumbar spinal MRI.

## 1. Introduction

Disc herniation and vertebral injuries have become increasingly common among youth [1]. Magnetic resonance imaging (MRI) of the lumbar spine plays an important role in diagnosing these conditions. Segmentation of these spinal structures helps expedite the diagnosis process, making for a better patient outcome. Spinal MRI segmentation has typically used manual labeling or semi-automated methods like active contouring, which are slow and vary between observers [2]. Developing fully automated and precise segmentation tools for T1-weighted spinal MRIs remains an important need to streamline the diagnostic workflows and clinical outcomes. Despite the availability of segmentation algorithms, T1-weighted lumbar spine MRIs present a different set of challenges. Unlike the traditional T2 images, T1 sequences are much less frequently targeted for segmentation because of the low contrast resolution, complicating different spinal structures [3]. Compared to T2-weighted MRI segmentation, deep learning approaches for T1-weighted lumbar spine imaging remain relatively underexplored. T1-weighted MRI segmentation is also difficult because variable spin-echo and gradient-echo parameters cause inconsistent tissue contrast and boundaries across scans, hindering deep learning accuracy [4,5,6].

*The benefits of T1-weighted lumbar spine segmentations*: T1-weighted lumbar spinal segmentations provide insight into bone marrow composition, vertebral structure, and degenerative changes [7]. This is not as apparent on T2-weighted images, making T1-weighted imaging especially useful for osteoporotic fractures and metastatic lesions, where the loss of the normal high marrow fat signal on T1 provides a clearer indication of bone marrow infiltration or collapse [8,9]. T2 sequences remain the gold standard for spinal MRI segmentation, but T1′s ability to highlight fat and bone-specific characteristics makes them important in many clinical workflows [7]. The goal of this paper was to build a novel tool to help orthopedic surgeons and radiologists expedite the diagnostic workflow. In this work, we benchmark an Attention U-Net–based segmentation pipeline against multiple deep learning architectures specifically for lumbar T1-weighted spinal MRI. Our contribution lies in performing a comparative evaluation coupled with radiologist validation, demonstrating that the Attention U-Net achieves the best clinical reliability and segmentation quality among state-of-the-art methods.

*Deep learning in spinal MRI:* Deep learning has emerged as a powerful tool in radiology. It has enabled highly accurate and efficient segmentations of different spinal structures. Among the most widely used techniques are convolutional neural networks (CNNs) which excel in recognizing the complex patterns in medical images, even in cases when the image quality is not the best [10]. One of the most significant advantages of deep learning in spinal segmentation is its ability to work across a large range of imaging modalities like MRI, CT, and X-rays. For our case, this robustness is important for the wide range of patient spinal scans. For instance, U-Net and its variants are commonly applied to 2D and 3D MRI datasets for T2-weighted segmentations, effectively delineating the vertebrae and vertebral discs with high precision [11]. V-nets and other volumetric CNN models are important for CT, as they can analyze the entire 3D volumes in a single step [12]. However, these algorithms take a much longer time to process the full spinal segmentation volume. Two-dimensional U-Nets are a medium between the deep learning and the processing power. They produce quick segmentations and can iterate in 3D if necessary. Hybrid models are often used for complex cases like fused vertebrae in scoliosis [13]. They were not adopted because of their computational complexity and slower interference times, which would hinder efficient clinical deployment.

*Other Tools for Segmentation:* While deep learning methods have garnered attention for T2 spinal segmentation tasks, other algorithms have also demonstrated success. For example, an atlas-based segmentation has been a reliable method to segment lumbar spines in T2-weighted MRI [14]. This technique consists of a pre-labeled registered atlas with a template of the patient’s imaging data. The anatomical features are then aligned with deformation fields. The issue with this approach is that it is computationally challenging. However, atlas-based methods have shown exceptional performance in delineating spinal structures. Active contour models have also been used for spinal segmentation in T2-weighted images, where there was a noticeable contrast between the spinal structures and surrounding tissues [15]. Active contour methods have achieved a strong accuracy in identifying abnormal spinal regions like herniated discus or regions with spinal stenosis. Support vector machines and random forest classifiers have also added to T2-weighted segmentation tasks. These algorithms work by using handcrafted features like intensity gradients, texture, and sharp descriptors to differentiate spinal structures. SVMs have been applied for segmenting vertebral bodies and discs in smaller datasets. Deep learning is not used, as it may over fit.

*The U-Net Architecture*: The infamous U-Net architecture has arguably become one of the most effective DL models for medical image segmentation tasks, excelling in T2 spinal MRI segmentation [16,17]. It is effective because of its encoder–decoder structure that has skip connections linking corresponding layers, allowing the model preserve crucial spatial information during reconstruction. This is important for segmenting spinal structure in T2-weighted MRI. Another critical component of the U-net is its ability to handle small datasets. This is a common limitation in medical imaging and another important reason for its success. The symmetry of the architecture on reliance on data augmentation can boost accuracy even in the cases of limited training dataset. The 3D U-Net also shows promise in volumetric segmentation; however, the computational resources needed for this is immense [18]. Two-dimensional U-Nets enable fast segmentation of spinal structures but may need to be improved to deal with the spinal T1-MRI landscape. Variations of the U-net remain the gold standard for segmentation tasks, especially in segmentation. Thus, in this study, we focus on altering the U-Net. The rationale for choosing an attention-based segmentation model lies in its architectural design, which integrates attention gates within the skip connections of the U-Net. These gates allow the network to suppress irrelevant background features and enhance the representation of spinal boundaries, improving the segmentation accuracy on low-contrast T1-weighted images.

## 2. Materials and Methods

### 2.1. Dataset

In this study, we used a publicly available lumbar spine MRI dataset by van der Graaf et al. [19]. This was a multi-center dataset that consisted of 218 patients with a history of low back pain collected from four different hospitals in the Netherlands. Each patient’s metadata included T1-weighted, T2-weighted, and T2 space sequences. During the experiments, we focused primarily on making a T1-weighted algorithm; thus, the other sequences were discarded. The dataset consisted of 477 sagittal MRI series with 3125 vertebrae and 3147 intervertebral discs. The gender distribution was 63% female and 37% male. Image resolutions were 0.90 × 0.47 × 0.47 mm. The segmentation maps that were collected were from a combination of manually annotated and semi-automatic algorithms. These maps were collected as part of the dataset and used as the ground truth [19]. Each of the 3D volumes consisted of (512, 512, 16) matrices. Algorithms were trained on the middle slice (8th slice) or a combination of middle slices (6–11 indices). The final matrices were input into the algorithm as (512, 512). Maps segmented the vertebral bodies and the spinal canal. There were different classes, but the main ones used in this study were 0 vs. 1, as there was no discrimination between the spinal cord and vertebrae.

All MRI studies were de-identified and released under a CC-BY 4.0 license following institutional review board approval at Radboud University Medical Center (IRB 2016–2275). The scans were acquired using multiple MRI scanners and protocols across four hospitals, introducing natural variability in voxel spacing and contrast that enhances model generalizability (Siemens Healthineers, Erlangen, Germany; Philips Healthcare, Best, The Netherlands). Each segmentation was reviewed by an expert musculoskeletal radiologist to ensure the consistency and accuracy of vertebral and disc boundaries.

### 2.2. Attention U-Net

To best segment small spinal structures and the spinal cord, we hypothesized that the Attention U-Net (AttU-Net) would perform better than the other variations of the U-Net. Unlike the traditional U-Net, which relies largely on the skip connections to concatenate encoder and decoder features, the AttU-Net introduces attention gates. These gates allow the network to focus more on relevant information while suppressing some of the irrelevant background, boosting the segmentation accuracy.

**Figure 1:**
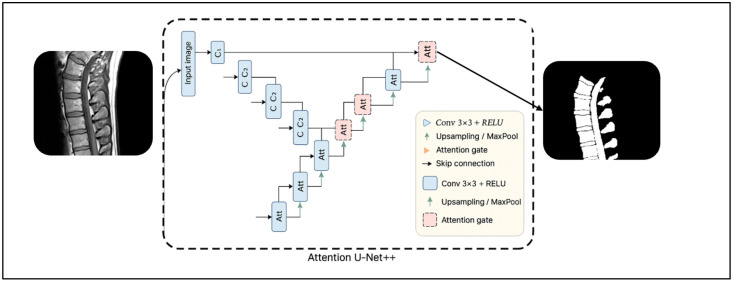
The Attention U-Net architecture for spinal MRI segmentation. The model extends the standard U-Net by integrating attention gates along skip connections to focus on relevant spinal regions while suppressing background noise. It takes an MRI slice as input and produces a refined segmentation map highlighting key spinal structures [20].

The attention U-Net follows the classical encoder–decoder pathway of the general U-Net (Figure 1). The encoder consists of several convolutional layers, which are followed by downsampling operations through the max pooling function. The decoder has upsampling layers that are concatenated with the corresponding encoder features through skip connections. The processes can be easily shown in Equation (1):

(1) $$G^{l}=f_{dec}^{l}(Up\left(G^{\;l\;+\;1}\right)⊕F^{l}.$$

The sequential convolutional layers are followed by downsampling the maxpooling layers which produce feature maps F^l^ at each l layer. The decoder performs the upsampling operation that combines the upsampled decoder features represented by G^l^ + 1, which the encoder features using F^l^ skip connections. The ⊕ represents the concatenation, and the Up(.) is the upsampling operation where $f_{dec}^{l}$ is the convolution for the decoder. The attention gate is the key innovation in the attention U-Net and is what differentiates it from the other algorithms. Specifically, it improves the skip connections by attempting to highlight the relevant encoder features before they are combined with the decoder features. First, the inputs are linearly transformed using 1 × 1 convolutions to align their dimensions. $W_{x}$ and $W_{g}$ are the learned weights during the training process.

(2) $$\psi =ReLU\left(W_{x}F_{l}+W_{g}G^{l+1}\right)$$

The attention map, α, is created when the intermediate map, ψ, is passed through a sigmoid activation function:

(3) $$\alpha =\sigma (W_{\psi }\psi ).$$

The attention map is then applied to the encoder feature map, Fl, using an element wise multiplication function:

(4) $$F_{att}^{l}=F^{l}\;*\;\alpha .$$

The fully refined encoder features Flatt are concatenated with the upsampled decoder features:

(5) $$G^{l}=f_{dec}^{l}(Up\left(G^{\;l\;+\;1}\right)⊕F_{att}^{l}.$$

The Attention U-Net was optimized to improve the sensitivity to subtle vertebral boundaries in low-contrast T1-weighted MRI. The architecture consisted of five encoder–decoder stages with channel depths from 64 to 1024, allowing progressive extraction of both shallow and deep anatomical features. Skip connections were enhanced using attention gates tuned to half the channel dimensionality (e.g., F_int_ = ½ F_g_), which selectively amplified the spinal canal and vertebral features, while suppressing soft tissue noise. Each convolutional block used batch normalization and ReLU activations to stabilize the feature propagation across layers, while up-convolution layers maintained spatial coherence during reconstruction. We initialized all layers using Kaiming normalization and refined kernel sizes and activation functions to improve the convergence on grayscale T1 data. These architectural optimizations improved the feature localization and segmentation consistency across variable contrast levels inherent to multi-center T1 MRI scans.

#### Model Learning and Hyperparameters

The model training was conducted using a random split 70:30 of the training set and the testing set. In this study, our priority was a simple and computational effective approach. Thus, when we were choosing the loss function, we opted for the one that would train our model the fastest. After experimenting with multiple approaches, we decided to use mean squared error (MSE) loss. This loss, which is commonly used in regression tasks, calculated the average of the squared difference between the output and the ground truth, making it an effective loss function for pixel-wide comparison in segmentation tasks like the one in this study. One of the reasons we selected MSE loss over other more commonly used losses like binary cross entropy (BCE) was that the BCE did not yield satisfactory segmentation outputs during our initial experiments. Specifically, when we applied BCE loss in the first iteration of our model training, segmentation results were very inconsistent and did not capture the standard boundaries of the spinal structures. As mentioned in the dataset portion, the model training took in slices of (512, 512) instead of the full volumetric slices. This significantly sped up the training time and allowed for more optimization. All training was completed on PyTorch 1.7.0 using (hardware, e.g., GPU, etc.) Northwestern’s high performance computing cluster, Quest. Model training and evaluation were performed using the mean squared error. The Adam optimizer was used for gradient descent. The learning rate was set to 1 × 10^−4^, and the weight decay was set to 5 × 10^−4^. The batch size was 1.

To ensure reproducibility, the training and testing datasets were separated at the subject level to prevent any overlap between slices from the same individual. Each 3D MRI volume was decomposed into axial 2D slices, which each served as independent samples for model training. Prior to input, all slices were resized to 256 × 256 pixels to standardize the resolution across subjects and scanners. The model weights were initialized using Kaiming normal initialization to promote stable gradient propagation during early training. To enhance generalization, random horizontal flips (*p* = 0.5) and random in-plane rotations (±10°) were applied as data augmentations. Each image slice was normalized to zero mean and unit variance prior to training. All training was performed on a CUDA-enabled NVIDIA GPU (NVIDIA Corporation, Santa Clara, California, United States) within Northwestern’s Quest high-performance computing cluster for a total of 100 epochs. No cross validation or early stopping was applied; model performance was evaluated on the held-out testing set after full training.

### 2.3. Radiologist Study

To evaluate the clinical relevance of the model outputs, two board-certified neuroradiologists independently participated in a blind scoring study. Each radiologist assessed five randomly selected lumbar spinal MRI segmentations per network, using the corresponding ground truth masks as reference. All scans were consistent across models to ensure a fair comparison. Radiologists rated the segmentation quality on a 5-point Likert scale (1 = poor, 5 = excellent) based on image quality and anatomical accuracy, and each indicated their preferred model overall. Inter-rater reliability was evaluated using Cohen’s Kappa coefficient.

## 3. Results

### 3.1. Structural Similarity + DICE Scores

**Figure 2:**
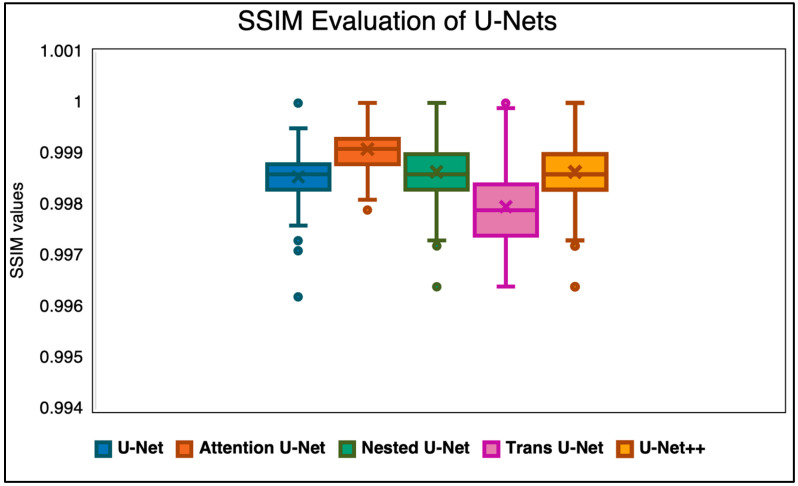
SSIM evaluation of the Attention U-Net against other state-of-the-art algorithms.

The first experiment we conducted was to compare whether the attention U-Net would perform competitively against the other state-of-the-art U-Nets. These algorithms were chosen based on their prior performance in segmentation tasks within brain MRI and spinal MRI. The first evaluation in this category was SSIM (Figure 2). The SSIM metric was used to determine whether the overall similarity and pixel intensity was the same. Intensity values are critical in determining disc herniations or spinal stenosis diagnoses. Subtle variations in this pixel intensity can ultimately indicate the presence of structural abnormalities or inflammation. By looking at the SSIM, we aim to check the model’s ability to preserve anatomical detail and also capture the intensity differences. Deep learning models are sometimes susceptible to changing the intensity distribution. Monitoring that component is critical in an accurate diagnosis.

**Figure 3:**
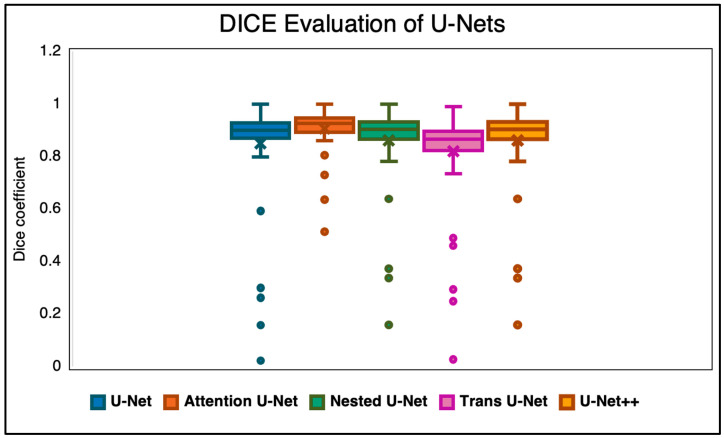
DICE evaluation of Attention U-Net against other state-of-the-art networks.

The second quantitative metric we used to evaluate the outputs of the network was the DICE score (Figure 3). While the SSIM does evaluate the overall structural and intensity similarity between the GT and the predicted values, the DICE coefficient measures the overlap of the spinal regions (ROIs) against the ground truth segmentation masks. This is a widely used metric to determine how well the predicted segmentation aligns with the ground truth by calculating the ratio of twice the intersection area to the total combined area of both sets. Higher DICE scores, naturally, indicate better agreement, with a perfect score being 1. The same set of algorithms were used to evaluate the similarity for DICE as with the SSIM.

### 3.2. Radiologist Clinical Assessment

**Figure 4:**
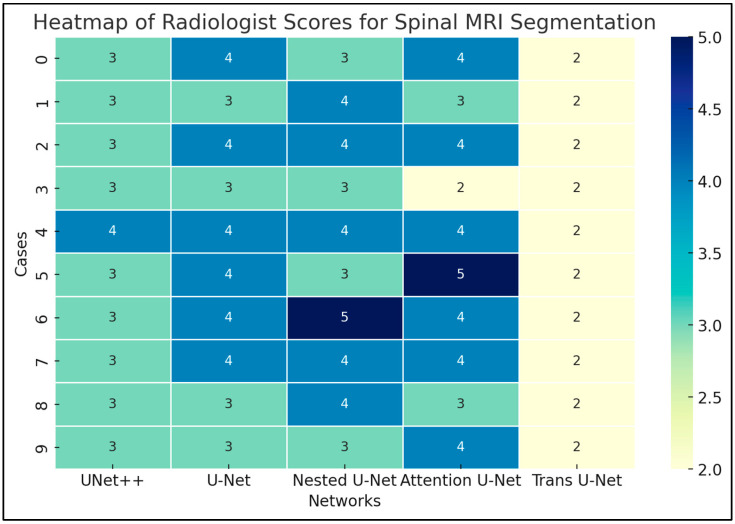
Radiologist score heatmap of the Attention U-Net against other state-of-the-art models. The stronger blue represents a better score, and a lighter yellow represents a worse score. Intensity distributions are combined from both radiologists.

Radiologist assessment provided additional clinical validation for the segmentation models. Both board-certified neuroradiologists identified the Attention U-Net as the top-performing model, noting its superior boundary delineation and anatomical accuracy compared to other architectures. The ratings between the two radiologists showed fair agreement (κ = 0.31), and the regression analysis indicated similar scoring trends between raters (Figure 4, Table 1). These findings suggest that, despite modest inter-rater variability, the Attention U-Net produced the most consistent and clinically reliable segmentation outputs.

### 3.3. Qualitative Comparison

**Figure 5:**
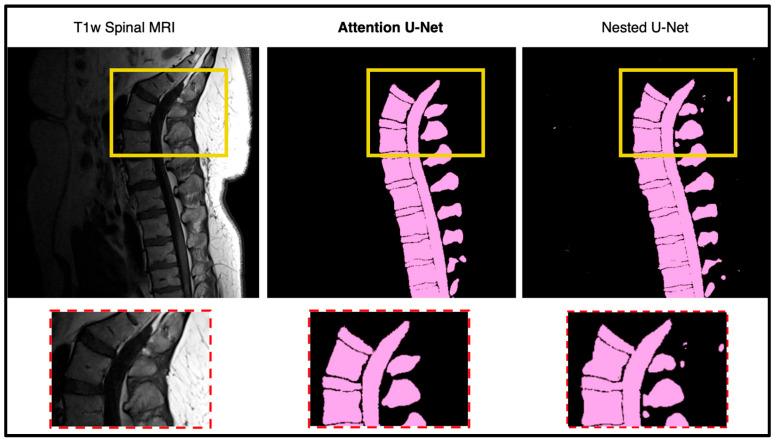
Qualitative comparison between Attention U-Net and Nested U-Net segmentation outputs on a lumbar T1-weighted spinal MRI. The highlighted regions show that Attention U-Net produces cleaner and more anatomically consistent boundaries, accurately following vertebral contours. In contrast, Nested U-Net introduces small artifacts and irregularities in adjacent soft tissue regions, indicating reduced boundary precision.

**Figure 6:**
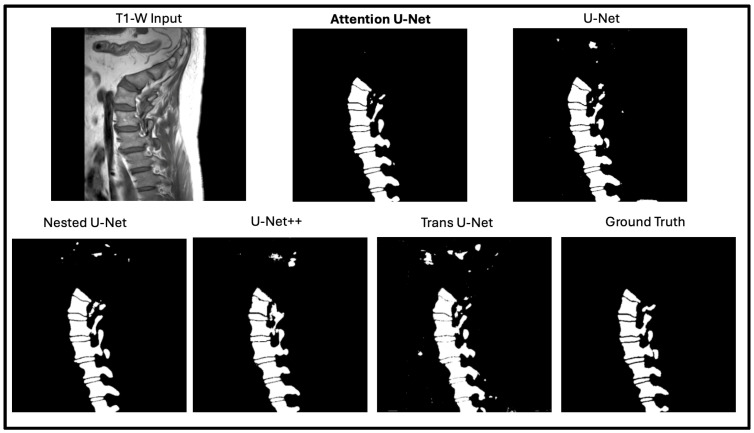
Segmentation comparison across five U-Net variants on a lumbar T1-weighted spinal MRI. The Attention U-Net produces the most anatomically accurate result, closely matching the ground truth with clear vertebral boundaries. Other models show minor artifacts or boundary inconsistencies, highlighting the Attention U-Net’s stronger feature localization and structural precision.

**Figure 7:**
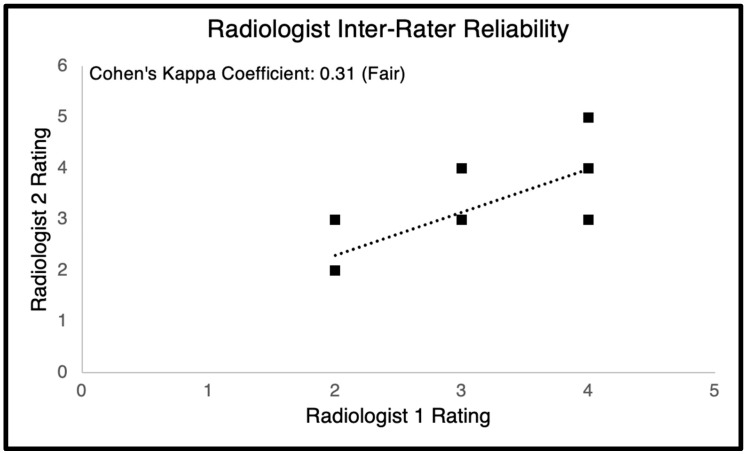
Scatter plot showing inter-rater reliability between two board-certified radiologists evaluating model outputs. The Cohen’s Kappa coefficient of 0.31 indicates fair agreement, reflecting moderate consistency in subjective image quality assessment. Variability in scores likely stems from differing radiologist focus areas and interpretation criteria during segmentation evaluation.

In this portion of the results, we focus on manually assessing the segmentation quality of the network outputs (Figure 5). While quantitative metrics like DICE and SSIM capture the quantitative performance of the segmentation models, we still wanted to see how these outputs are reflected in real time. DICE and SSIM might fail to highlight some subtle boundary mismatches or some issues in segmentation of complex structures. By incorporating qualitative observations, we aim to provide a more comprehensive evaluation of the network’s performance. Figure 6 shows the output of the model against the four other state-of-the-art U-Net networks. The attention U-Net is bolded as the top algorithm. Two algorithms that were close with respect to DICE, SSIM, and the radiologist reports were the Attention U-Net and the Nested U-Net. We thought it would be best if we showed the visualization to demonstrate the differences. Figure 7 highlights the difference and clearly shows the addition artifacts created by the Nested U-Net. While most of the segmentation is similar, the edges are not as clearly defined. The red boxes show the zoomed in portion of both, further demonstrating the difference between the two.

### 3.4. Loss Ablation Studies

**Figure 8:**
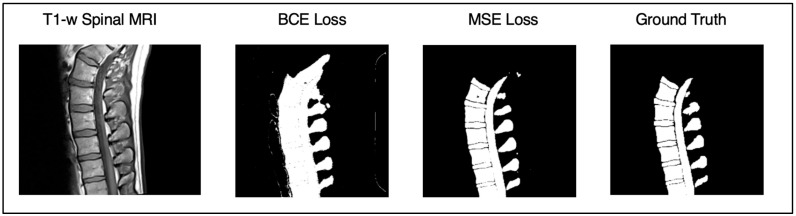
Comparison of segmentation outputs from the Attention U-Net trained with binary cross-entropy (BCE) and mean squared error (MSE) loss on a representative lumbar T1-weighted spinal MRI. The BCE loss output shows boundary blurring and partial fusion of adjacent vertebrae, while the MSE loss preserves sharper and anatomically consistent edges. The MSE-based segmentation most closely matches the manual ground truth, demonstrating improved precision in delineating spinal structures.

**Figure 9:**
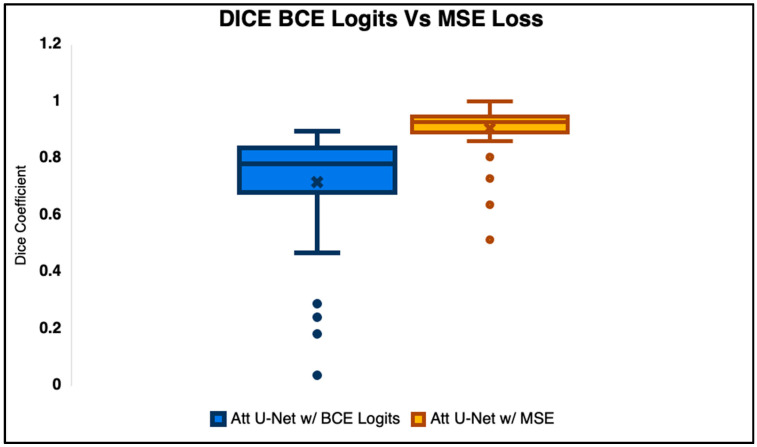
Comparison of segmentation performance for Attention U-Net using binary cross entropy (BCE) with logits loss versus mean squared error (MSE) loss. The left boxplot shows DICE coefficients, where the MSE loss achieved higher median and more cons.

Binary cross entropy loss (BCE) is a standard metric used in segmentation tasks, especially in brain MRI analysis. During this portion of the experiment, we wanted to see how the BCE with logits loss would compare to the MSE loss. The primary assessment focused on the quantitative metrics that were used to evaluate the Attention U-Net against the state of the art: DICE and SSIM. Radiologists did not score the outputs of the BCE vs. MSE, as this was hyperparameter tuning, and it was very clear which algorithm was superior upon visual examination (Figure 8). The results are presented using box and whisker charts, like the comparison against the state of the art. This shows how the data distribution is presented and highlights an accessible comparison. There is statistical significance between the MSE and BCE loss, which is reflected by the lack of substantial overlap in the SDs between the plots. In addition to these quantitative metrics, we also present visual comparisons of the segmentations to provide stronger context. These images highlight the impact of each loss function on the structural preservation of the segmentation maps, supporting the numerical data. Combining both the box and whisker plots and the visual comparisons provides a comprehensive depiction of the performance differences between the BCE and MSE loss functions (Figure 9). The outcome was also statistically significant (*p* < 0.05), with the MSE loss outperforming BCE loss in SSIM and DICE.

## 4. Discussion

The goal of this paper was to apply the attention U-Net algorithm towards automatic segmentation of lumbar T1-weighted spinal MRI images. The attention U-Net algorithm was hypothesized to be the strongest performing model and was the central neural network for the study. Firstly, the U-Net architecture is robust in medical image segmentation, offering robust segmentations for even the smallest medical cavities. The encoder–decoder structure maintains resolution, while incorporating contextual information from the decoder. The key innovation in the Attention U-Net is the attention gate mechanism. The gates are placed within the skip connections of the U-Net to help selectively filter the features that are passed from the encoder to the decoder. This allows for a more selective segmentation and reduces external noise.

To properly assess the Attention U-Net, we first started by quantitatively checking its accuracy. The first metric we employed was the SSIM. This metric helps to assess the structural similarity between the segmented image and the ground truth image. This was a baseline check to see if the models would maintain the same pixel-intensity distributions. These quantitative results confirmed that the Attention U-Net captures spinal structures more effectively than baseline U-Net variants, particularly in regions with subtle anatomical contrast. Although these improvements appear numerically modest, they may reflect meaningful gains in delineating vertebral margins, which are critical for downstream tasks such as spinal canal measurement or pathology detection. Compared with recent deep segmentation approaches such as nnU-Net or Swin-UNETR, our Attention U-Net achieves comparable structural fidelity while maintaining a lighter more computationally efficient design. The second quantitative evaluation technique we used was the DICE Coefficient. This metric measures the overlap between the segmented image and the ground truth. This metric is more accurate for clinical value. Although the Attention U-Net achieved slightly higher DICE and SSIM scores, the margin was modest and should be interpreted considering the dataset’s limited size. The other state-of-the-art algorithms performed lower than the Attention U-Net and the Nested U-Net with regard to both DICE and SSIM. These were the only two competitive algorithms.

Radiologist assessment helps prove the clinical worth of these algorithms. Two board-certified radiologists scored the outputs of the study on the Likert scale. It was found that the two best performing algorithms were the Nested U-Net and the Attention U-Net. The Attention U-Net, however, was the algorithm of choice for both radiologists. The scoring averages were similar for the attention U-Net and Nested U-Net, with the Nested U-Net performing slightly better in terms of the standard deviation. The radiologists were involved a blind study environment; so, no additional judgement could be made. The radiologists’ evaluations were to ensure the model’s performance was at least hitting the semi-correct target areas. The radiologist outputs were strong from both ends. A potential reason why the outputs was scored better can be seen from the qualitative observations. Unlike the Attention U-Net, the Nested U-Net generates artifacts in other regions of the MRI scan, where spinal mass is not generally found or was not present in the ground truth. This can offer explanations for why the Attention U-Net performed better in all the metrics. The Attention U-Net also has a smaller more specific scope, when segmenting lumbar cavities. This makes it a promising candidate for assisting radiologists, though further validation is needed before it can be considered clinically reliable. Radiologist inter-rater reliability was also calculated by running a regression line through the scores and then computing the Cohen’s Kappa. This coefficient explains how well the radiologists scores agreed with one another. The Cohen’s Kappa coefficient indicated a fair rating. This is as expected, because radiologists with differing expertise and differing locations may look at different parts of the spinal MRI when judging for accuracy. Although the radiologist validation added valuable clinical context, it was limited by the small number of raters and the small sample size of images evaluated. The fair inter-rater agreement suggests that subjective variability remains a challenge when interpreting segmentation quality. Future studies should include larger multi-reader cohorts and a broader range of spinal pathologies to more robustly assess clinical reliability and generalizability.

Although binary cross entropy (BCE) loss is a widely used and generally effective objective in segmentation tasks, our findings revealed notable advantages of using the mean squared error (MSE) loss for lumbar T1 spinal segmentation. While the BCE achieved strong baseline performance, the MSE consistently produced more precise delineations and clearer structural boundaries. Quantitatively, both DICE and SSIM scores were significantly higher for MSE-trained models (*p* < 0.05), indicating superior overlap accuracy and improved structural fidelity. The qualitative analysis further showed that the BCE often led to blurring at the edges of vertebral structures and partial fusion with surrounding tissues, which can be attributed to its sharp penalization of boundary misclassifications. In contrast, the regression-based formulation of MSE loss yielded smoother gradient propagation and greater sensitivity to local intensity variations, enabling the model to preserve fine anatomical details and maintain distinct vertebral contours.

With additional validation and regulatory approval, this algorithm could eventually support clinical workflows. This would ultimately help radiologists quickly identify key areas and reduce the time for manual labeling. Radiologist oversight would be necessary, however, for implementation in clinical practice, as the model has the potential to hallucinate. While there have been significant studies for T2, very few studies have involved T1-weighted images. This would be one of the first to target T1. The main novelty of this project is the use of the Attention U-Net specifically to spinal MRI structures. This applies attention gates to help focus on relevant areas and ignore background noise. FDA approval would also be necessary to fully activate this algorithm in clinical practice. More data would be needed to check whether the Attention U-Net can work with more diverse clinical cases.

Despite the strong segmentation performance of the Attention U-Net architecture, several limitations must be acknowledged. First, the model was trained on a relatively small and homogeneous dataset of a few hundred lumbar spine MRI scans, which limits its ability to generalize to different scanners, acquisition settings, or patient populations. This introduces potential dataset bias and increases the risk of overfitting, as the network may learn scanner-specific or demographic-specific features rather than universally robust representations. In addition, no external validation was performed on an independent dataset, which is essential to confirm the reproducibility and clinical applicability across institutions. The absence of simulated artifacts such as motion or intensity variation also limits the understanding of the model’s robustness in real-world imaging conditions. Future work should include extensive data augmentation and cross-domain testing to improve generalization and reduce bias. Furthermore, incorporating hybrid attention architectures such as transformer-based or channel and spatial attention modules and exploring regularization strategies may help stabilize training and enhance the performance consistency. Expanding the dataset and including multi-site and multi-reader evaluations will be important to strengthen the scalability and clinical reliability of the proposed framework.

## 5. Conclusions

In this paper, we introduced a deep learning framework for the automatic segmentation of lumbar T1-weighted spinal MRI scans using the Attention U-Net architecture. Our findings demonstrated that the Attention U-Net achieved the most consistent and accurate performance compared to other state-of-the-art segmentation networks, supported by both quantitative metrics and radiologist evaluations. The investigation of different loss functions further revealed that the mean squared error loss produced finer structural delineation than binary cross entropy in this specific context. Although the results are promising, the model remains a research prototype that requires further optimization, larger-scale validation, and multicenter testing to ensure generalizability and robustness across diverse clinical settings. Future work will focus on improving the model efficiency, expanding the dataset diversity, and developing an accessible platform to support broader research and clinical experimentation.

## Figures and Tables

**Table 1:** Radiologist Evaluation of Algorithms (Likert Scale).

UNet++	U-Net	Nested U-Net	Attention U-Net	Trans U-Net
3.1 ± 0.32	3.6 ± 0.52	3.7 ± 0.67	3.7 ± 0.82 *^,^ **^†^**	2.0 ± 0.0

* Preferred network by radiologist 1. **^†^** Preferred network by radiologist 2.

## Data Availability

The data presented in this study are openly available in Zenodo at https://doi.org/10.5281/zenodo.10159290 (accessed on 14 September 2024), reference number [14].
